# X-ray Absorption Near-Edge Structure (XANES) at the O *K*-Edge of Bulk Co_3_O_4_: Experimental and Theoretical Studies

**DOI:** 10.3390/nano12060921

**Published:** 2022-03-10

**Authors:** Stephane Kenmoe, Dick Hartmann Douma, Abdulrafiu Tunde Raji, Bernard M’Passi-Mabiala, Thomas Götsch, Frank Girgsdies, Axel Knop-Gericke, Robert Schlögl, Eckhard Spohr

**Affiliations:** 1 Department of Chemistry, University of Duisburg-Essen, Universitätsstr. 2, 45141 Essen, Germany; eckhard.spohr@uni-due.de; 2 Groupe de Simulations Numériques en Magnétisme et Catalyse, Faculté des Sciences et Techniques, Université Marien Ngouabi, Brazzaville B.P. 69, Congo; dick.douma@umng.cg (D.H.D.); bmpassimabiala@gmail.com (B.M.-M.); 3 Department of Physics, College of Science, Engineering and Technology (CSET), University of South Africa (UNISA), Corner of Christiaan de Wet Road & Pioneer Avenue, Florida 1709, South Africa; tunderaji@gmail.com; 4 National Institute of Theoretical and Computational Sciences (NITheCS), University of South Africa (UNISA), Preller St., Muckleneuk, Pretoria 0002, South Africa; 5 Unité de Recherche en Matériaux et Energies, Institut National de Recherche en Sciences Exactes et Naturelles, Brazzaville B.P. 2400, Congo; 6 Department of Inorganic Chemistry, Fritz-Haber-Institut der Max-Planck Gesellschaft, Faradayweg 4-6, 14195 Berlin, Germany; goetsch@fhi-berlin.mpg.de (T.G.); girgsdie@fhi-berlin.mpg.de (F.G.); knop@fhi-berlin.mpg.de (A.K.-G.); acsek@fhi-berlin.mpg.de (R.S.); 7 Department of Heterogeneous Reactions, Max Planck Institute for Chemical Energy Conversion, Stiftstraße 34-36, 45470 Mülheim an der Ruhr, Germany; 8 Center of Computational Sciences and Simulation, University of Duisburg-Essen, 45141 Essen, Germany

**Keywords:** X-ray absorption, cobalt tetraoxide, density functional theory, projector augmented wave method, dipole transition, *K*-edge spectrum, X-ray diffraction, Quantum-ESPRESSO

## Abstract

We combine theoretical and experimental X-ray absorption near-edge spectroscopy (XANES) to probe the local environment around cationic sites of bulk spinel cobalt tetraoxide (Co${}_{3}$O${}_{4}$). Specifically, we analyse the oxygen *K*-edge spectrum. We find an excellent agreement between our calculated spectra based on the density functional theory and the projector augmented wave method, previous calculations as well as with the experiment. The oxygen *K*-edge spectrum shows a strong pre-edge peak which can be ascribed to dipole transitions from O 1s to O 2p states hybridized with the unoccupied 3d states of cobalt atoms. Also, since Co${}_{3}$O${}_{4}$ contains two types of Co atoms, i.e., Co${}^{3+}$ and Co${}^{2+}$, we find that contribution of Co${}^{2+}$ ions to the pre-edge peak is solely due to single spin-polarized $t_{2g}$ orbitals ($d_{xz}$, $d_{yz}$, and $d_{xy}$) while that of Co${}^{3+}$ ions is due to spin-up and spin-down polarized $e_{g}$ orbitals ($d_{x^{2}-y^{2}}$ and $d_{z^{2}}$). Furthermore, we deduce the magnetic moments on the Co${}^{3+}$ and Co${}^{2+}$ to be zero and 3.00 $\mu_{B}$ respectively. This is consistent with an earlier experimental study which found that the magnetic structure of Co${}_{3}$O${}_{4}$ consists of antiferromagnetically ordered Co${}^{2+}$ spins, each of which is surrounded by four nearest neighbours of oppositely directed spins.

## 1. Introduction

Co${}_{3}$O${}_{4}$ nanoparticles are potential candidates to support many chemical reactions in heterogeneous catalysis [1,2,3,4]. This stems from its many beneficial electronic, redox and magnetic properties. To enhance the performance of nanoparticles and improve their rational design, fundamental insight into the key parameters such as coordination environment, spin and oxidation states of active sites is paramount. X-ray absorption near-edge spectroscopy (XANES) is an efficient technique to probe condensed matter at the atomic and subatomic levels and to determine information about the local environment of atoms. This is achieved by tuning the incident photon energy to the X-ray edge energy of the target atom of interest in the material.

For transition metal atoms that serve as active sites in many catalytic reactions, L${}_{23}$-edge X-ray absorption spectra (XAS) can be used to probe *3d* valence orbitals via the dipole-allowed 2p−3d transitions. However, from a theoretical point of view, obtaining a clear picture of the oxidation states from the L${}_{23}$-edge spectrum as calculated by density functional theory (DFT)-based single-electron approaches is not straightforward. This stems from the fact that the 2p-hole and 3d-hole radial wave functions in such systems overlap considerably. Besides, factors like the covalency of the metal-ligand bonds and the change in the metal oxidation and spin state may affect the spectral shape and incident energy [5,6,7]. All of these factors do not allow reliable comparisons of computed spectra with experimental ones. However, the ligand *K*-edge can be used to scan unoccupied 3d metal states, and to provide a more precise reproduction of the experimental data, as shown in recent DFT studies [8,9].

Recently, oxidation of 2-Propanol over the unsupported surface of Co${}_{3}$O${}_{4}$ spinel nanoparticles was studied using a variety of experimental techniques among which is the X-ray absorption spectroscopy (XAS) as well as the theoretical method of density-functional theory. The XAS study revealed the reduction of Co${}^{3+}$ to Co${}^{2+}$ during 2-propanol oxidation thus underlining the Co sites of Co${}_{3}$O${}_{4}$ surfaces as the active sites for the catalytic reactions [3]. Similarly, dissociation of water on two different Co${}_{3}$O${}_{4}$ (001) surface terminations was studied by Kox et al. [10]. Here, it was observed that water dissociates more frequently on the Co${}^{2+}$ sites than on the adjacent Co${}^{3+}$ sites, which again underlines different reactivity of the Co ions in the spinel Co${}_{3}$O${}_{4}$ surfaces. Thus, regarding catalytic reactions, it is important to probe the specific atomic site for its electronic properties which gives further insight into the magnetic and redox properties in a catalytic reaction. With respect to the Co${}_{3}$O${}_{4}$ nanoparticles in particular, in addition to Co ions, it is equally important to probe the electronic structure of O ion for completeness. Such a holistic approach is a major motivation in our research group, in our study of electronic, magnetic as well as catalytic applications of nanostructured Co${}_{3}$O${}_{4}$ surfaces. Thus, in the present work and as a step towards the understanding of effects of nanostructuring on the physical and chemical properties of the surfaces, including the influence of operando conditions on the local environment of Co active sites, we have performed combined experimental and theoretical studies of X-ray diffraction (XRD) and X-ray near-edge spectroscopy (XANES) of bulk Co${}_{3}$O${}_{4}$ to gain deeper insights into the bonding, coordination environment, spin and oxidation states of Co in bulk Co${}_{3}$O${}_{4}$. Our study provides a solid basis to analyze the interplay between electronic structure and catalytic reactions on nanostructured cobalt oxide catalysts in experimentally more relevant situations.

The outline of the paper is as follows: in Section 2, we provide details of XRD powder diffraction experiments performed to characterize the Co${}_{3}$O${}_{4}$ crystal phase. This section also contains experimental parameters for the X-ray absorption measurements as well as the details of computational XANES study. In Section 3, we present the results of the experimental and theoretical studies.

## 2. Experimental and Computational Details

### 2.1. Experimental Details

The experimental sample used for the X-ray absorption measurement consists of approximately 30 mg Co${}_{3}$O${}_{4}$ powder (Sigma Aldrich, (Saint Louis, MO, USA), ≥99.99%), which has been calcined at 873 K for 4 h prior to the measurements. The sample was subsequently pressed to a pellet (8 mm diameter) using a force of 30 kN. The X-ray measurements were performed at room temperature in a chamber that was back-filled to 0.5 hPa with N_2_ in order to enhance the collection efficiency of the total electron yield (TEY) signal. The spectrum was acquired at the soft X-ray branch of the EMIL beam line (UE48-soft PGM) at the BESSY II synchrotron, ref. [11] using the CAT (near-ambient pressure X-ray photoelectron spectroscopy (NAP-XPS)) end station. The TEY signal was obtained by collecting all released electrons at the entrance aperture of the differentially pumped hemispherical sector analyzer (Specs Phoibos 150 NAP), which was biased to 90 V.

**Figure 1:**
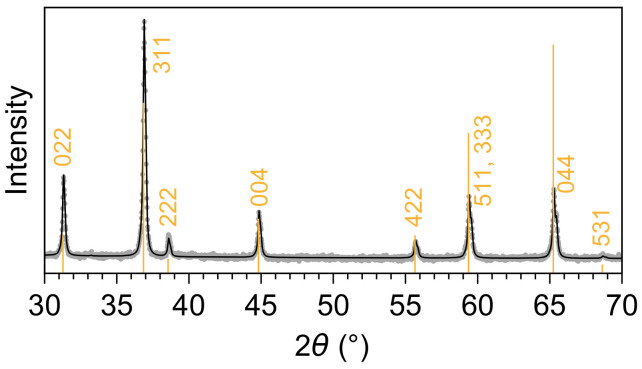
XRD and Rietveld fit for the cobalt oxide specimen used in the experimental study. The diffractogram can be described fully by the spinel oxide Co${}_{3}$O${}_{4}$ with a lattice parameter of 8.083 Å [12].

The phase purity of the Co${}_{3}$O${}_{4}$ sample was determined by X-ray diffraction (XRD) measurements. These were performed in Bragg-Brentano geometry on a Bruker AXS D8 Advance II theta/theta diffractometer, using Ni-filtered Cu K${}_{\alpha 1+2}$ radiation and a position-sensitive energy-dispersive LynxEye silicon strip detector. The diffractogram in Figure 1 shows that the spinel oxide is of a pure phase and only contains contributions from Co${}_{3}$O${}_{4}$.

### 2.2. Computational Details

**Figure 2:**
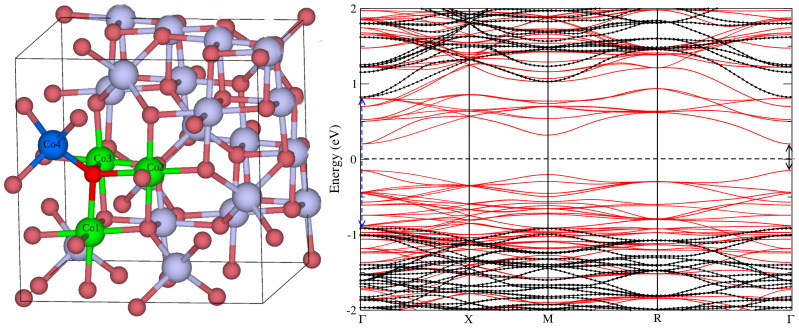
Co${}_{3}$O${}_{4}$ crystal structure and the corresponding bands calculated with the standard GGA (red lines) and GGA + *U* (black lines) respectively. In the crystal structure, the bluish grey and red spheres represent Co and O atoms, respectively. The bright red sphere represents the photon-absorbing oxygen atom bonded to the cobalt atoms in the local octahedral (green) and tetrahedral (blue) environments. In the bands, the dashed vertical arrow and the solid arrow show the electronic band gaps as obtained with the GGA + *U* and the GGA functionals, respectively. The arrows are drawn at the band edges which are located at the Γ point of the Brillouin zone.

All the DFT calculations have been performed using the Quantum-ESPRESSO (QE) software package [13] and with the calculation parameters described below. The conventional unit cell Co${}_{3}$O${}_{4}$ used in the calculations is shown in Figure 2. It consists of 56 atoms with an optimized lattice parameter of a=8.16 Å, which is in a good agreement with the experimental lattice parameter of 8.08 Å, as determined from the XRD (Figure 1) and as reported in a previous experiment [14]. Also, the Co${}_{3}$O${}_{4}$ crystal structure is a normal spinel structure (space group Fd$\bar{3}$m) having two types of sites for the Co atom- the tetrahedral (8*a*, blue sphere) and the octahedral (16*d*, green sphere) of the closed-packed face-centred cubic (FCC) lattice. On the tetrahedral site is the Co atom having +3 oxidation state, i.e., Co${}^{3+}$ while the octahedral Co has Co${}^{2+}$ state [14]. In the optimized Co${}_{3}$O${}_{4}$, the Co${}^{2+}-$O and Co${}^{3+}-$O separations are about 1.98 Å and 1.93 Å respectively. With regards to the calculation parameters, ultrasoft pseudopotential (USPP) [15] was used to describe the interactions between the ion cores and valence electrons. The Co and O USPPs have been obtained from the QE pseudopotential repository [16]. Calculations were performed with ordinary generalized gradient approximation (GGA) as well as the GGA + *U* where *U* is the so-called Hubbard correction. In the case of Co${}_{3}$O${}_{4}$, the *U* serves to exclude the self-interaction in the localized Co *d* electrons in the GGA approximation. We have used the GGA functional of Perdew-Burke-Ernzerhof (PBE) variant. Also, we have adopted the rotationally invariant formulation of *U* and an on-site *U* = 3.5. It should be noted that *U* has been used to correct the band gap in oxide materials and in particular, *U* of between 3.3 and 3.5 eV has been shown to give the correct band gap as well as physical and magnetic properties of cobalt oxides (including Co${}_{3}$O${}_{4}$), in good agreement with the experimental data [17]. Furthermore, structural optimization of the atomic positions was performed using the Broyden-Fletcher-Goldfarb-Shanno (BFGS) quasi-Newton algorithm gradient method with force convergence threshold of $10^{-5}\;\mathrm{eV}/Å$. Other calculation parameters employed to achieve convergence are a plane wave cut-off energy of 40Ry and a 2 × 2 × 2 *k*-point mesh [18] sampling of the Brillouin zone. However the density of states calculations were performed with a denser *k*-grid of size 6×6×6.

The XANES spectra have been obtained using the XSPECTRA code [19] which is a module in the QE software package. Therein, the X-ray absorption cross-section is expressed in terms of a transition operator coupling the initial and the final states which are solutions of the Kohn-Sham (KS) equations. In the case of the *K*-edge, the initial state is a core 1*s* orbital obtained from an isolated absorbing atom in the absence of a core hole (it is extracted from the oxygen GIPAW pseudopotential O.pbe-van_gipaw.UPF), while the final state is obtained self-consistently through the resolution of the KS equations for the whole system, including core hole effects in the pseudopotential of the absorbing atom [19]. Within pseudopotential approach, the final all-electron wave function is reconstructed from the pseudowave function by mean of Projector Augmented Wave (PAW) method [20]. The oxygen Gauge-Including PAW (GIPAW) pseudopotential which has a core-hole in the 1*s* state (i.e., O.star1s-pbe-van_gipaw.UPF), has been obtained from the QE website [16]. The mathematical basis of X-ray absorption spectroscopy in the USPP scheme and the use of the PAW method to reconstruct the all-electron wave function, and to obtain the XANES intensities have been described in the reference [19]. Furthermore, the isotropic electric dipole cross-section is obtained by a linear combination of the three XANES cross sections calculated along the three perpendicular directions of polarization as $\sigma \left(0,0\right)=\frac{1}{3}\left(\sigma_{xx}+\sigma_{yy}+\sigma_{zz}\right)$ [21]. In practice, the cross-section is calculated for a given polarization direction using the Lanczos algorithm and the continued fraction method [22]. This approach does not require an explicit calculation of empty states and is very fast, since only the charge density is needed [19]. A Lorentzian convolution with a variable broadening parameter γ has been applied in the continued fraction method to reproduce the experimental spectrum for the bulk Co${}_{3}$O${}_{4}$. It should be noted that generally, experimental X-ray absorption spectra contain fewer structures and display broader features than theoretical spectra. This is due to the fact that the finite lifetime of the core-hole is usually neglected in the theoretical calculations. Thus, to facilitate proper comparison between theory and experiment, the calculated spectrum is modified so that the finite core-hole lifetime is accounted for. A convenient way that is often employed to achieve this is to convolute the raw spectrum a posteriori with a Lorentzian [23]. For this purpose, we used γ=0.3 eV for the incident photon energy of up to 1.5 eV and γ=0.8 eV above a photon energy of 10 eV while γ varies linearly in the intermediate photon range between the two photon energy values.

## 3. Results

**Figure 3:**
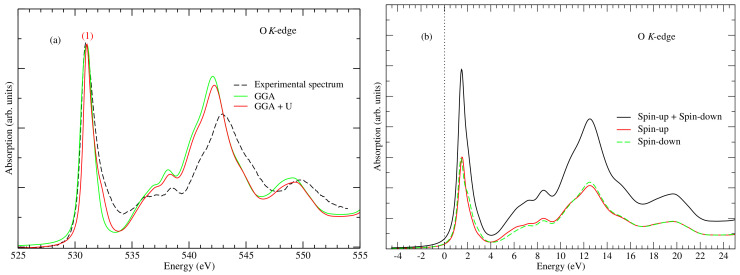
(**a**) O *K*-edge XANES spectrum calculated with the standard GGA functional (green line) and GG + *U* (red line) and the room temperature XAS spectrum recorded in TEY mode (black dashed line) for the Co${}_{3}$O${}_{4}$. (**b**) Spin-polarization dependence of the O *K*-edge XANES spectrum calculated with GGA + *U*, where the photon-absorbing oxygen atom is bounded to the cobalt atoms Co1, Co2, Co3 and Co4 as shown in the Co${}_{3}$O${}_{4}$ crystal in Figure 1.

The structural parameters of the model Co${}_{3}$O${}_{4}$ used for the XANES calculations have been stated in Section 2.2. Also, our calculations parameters, in particular GGA + *U* with *U* = 3.5 eV, gives a band gap value of 1.72 eV in excellent agreement with the experiment band gap E${}_{g}$ = 1.60 eV [24,25]. Ordinary GGA (i.e., without the inclusion of *U*) gives E${}_{g}$ = 0.34 eV. This is consistent with the well-known shortcomings of GGA and the LDA functionals, i.e., an underestimation of the bandgap in semiconductors [26,27]. The GGA + *U* is computationally frugal while providing a better description of the electronic structure of Co${}_{3}$O${}_{4}$ relative to the ordinary GGA, as has been shown for similar oxide materials [28]. The right-hand panel of Figure 2 shows the band structure of Co${}_{3}$O${}_{4}$ showing the clear difference between the GGA and the GGA + *U* bands and the relative accuracy of the latter. Nevertheless, we have performed the calculations of the XANES spectra calculation with both the GGA and GGA + *U* to determine the possible effect of the *U* on the spectra. Figure 3a shows the comparison between the experimental and computed oxygen *K*-edge XANES spectra. We observed no major discrepancy between the calculated spectra, in particular the positions and the intensity of the peaks are similar. Therefore, henceforth, our analysis will be based on spectra obtained with the GGA + *U*. In Figure 3b, spin-polarized O *K*-edge XANES spectra are presented. These spectra have been obtained by averaging the XANES O *K*-edge of all oxygen atoms in the elementary cell. Firstly, we observe good agreement between the computed and experimental spectra in that they have a common intense pre-edge peak at about 531 eV (marked as peak (1) in the figure). Also, our XANES spectra (computed and calculated) are also consistent with earlier studies by van Elp et al. [29], of the electronic structure of Co${}_{3}$O${}_{4}$ crystal as obtained via the X-ray absorption studies and model cluster calculations. Beyond the pre-edge peak, there is also a good agreement between the position of the major peaks save for a small relative shift. Also, as shown in Figure 3b, the spin-up and spin-down XANES spectra are similar and their superposition results in the calculated XANES O *K*-edge spectrum. Thus, the XANES spectra are not spin-dependent.

**Figure 4:**
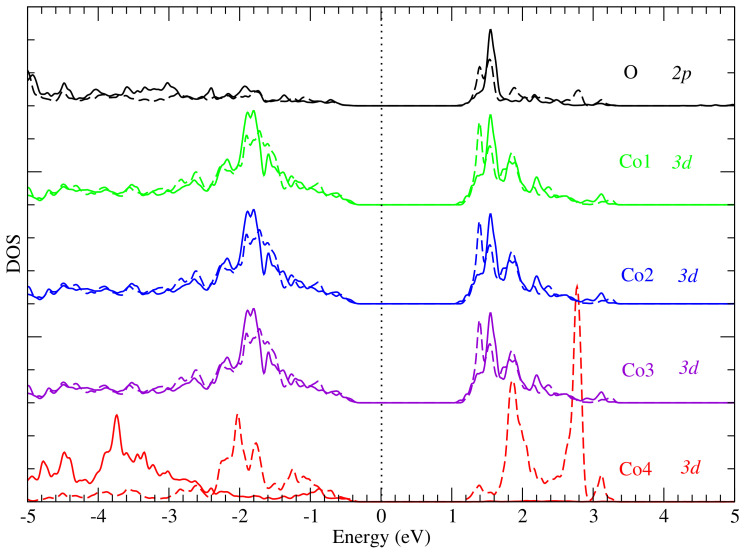
Partial DOS, for the 2p orbital of the absorbing oxygen atom and 3d orbitals of the nearest cobalt atoms Co1, Co2, Co3 and Co4. Solid and dashed lines correspond to spin-up and spin-down DOS components, respectively.

To gain deeper insight particularly into the origin of the pre-edge peak, we considered four cobalt atoms Co1, Co2, Co3 and Co4 (see Figure 2), which are bonded to the photon-absorbing oxygen atom. The cobalt atoms Co1, Co2 and Co3 have octahedral geometries (local point group $O_{h}$) while Co4 has a tetrahedral geometry (local point group $T_{d}$). For these atoms, we have plotted the partial densities of state (PDOS) separately in Figure 4. From this figure, one notices the similarity in the PDOS of Co1, Co2 and Co3, which is expected, since their local environment has the same symmetry relative to the O atom. Also, the PDOS plot shows a strong hybridization between the unoccupied Co 3d and the oxygen 2p states (above the Fermi level). When compared to the XANES spectra (i.e., Figure 3a), this energy range of hybridization corresponds to between 530 and 534 eV in Figure 3a. Thus, the pre-edge peak can be ascribed to dipole transitions from O 1s to O 2p states with the latter being hybridized with the unoccupied 3d states belonging to cobalt atoms Co1, Co2, Co3 and Co4, since they are bonded to the photon absorbing oxygen atom. Furthermore, the hybridization is stronger for the cobalt atoms Co1, Co2 and Co3 than for Co4, as shown by the PDOS plots in Figure 4. A possible explanation for this observation is that the O−Co4 bond is slightly longer than the other three Co−O bonds [29] which may suggest a smaller degree of bonding and thus hybridization between the oxygen and Co4 atom.

**Figure 5:**
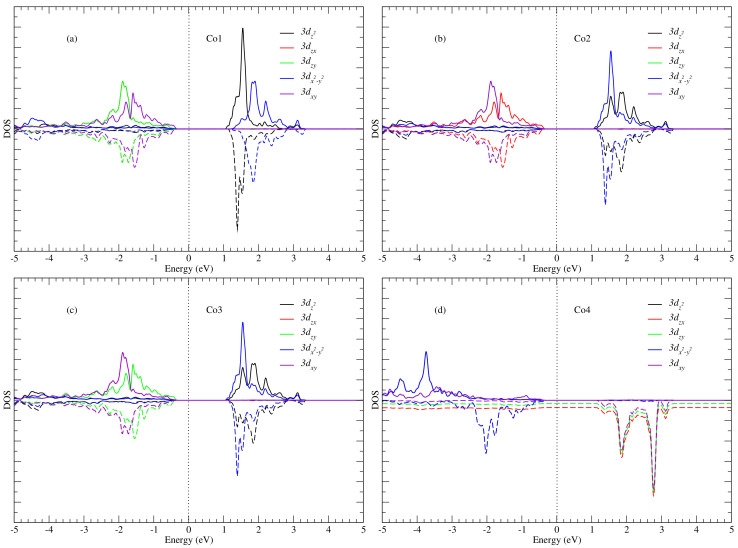
Partial DOS, for the 3d orbitals of the nearest cobalt atoms Co1 (**a**), Co2 (**b**), Co3 (**c**) and Co4 (**d**) to the oxygen absorbing atoms. Solid and dashed lines correspond to spin-up and spin-down DOS components respectively. The peaks located in the energy range between 0 and 3.4 eV are responsible for the pre-edge peak in the O *K*-edge spectrum between 530 and 534 eV (Figure 3).

**Figure 6:**
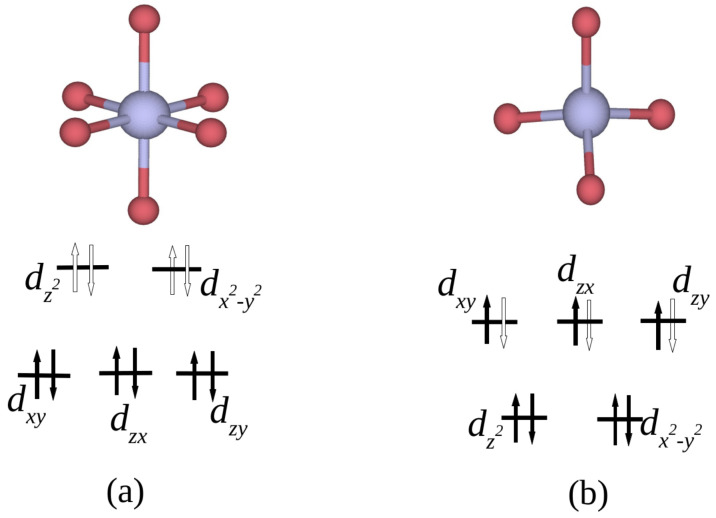
Cobalt geometries and the corresponding crystal-field splitting diagrams. Black filled arrows represent occupied electron states, while the hollow arrows characterize empty electron states. Bluish grey and red spheres represent Co and O atoms, respectively. (**a**) Co${}^{3+}$ ion in the octahedral geometry (representative for Co1, Co2 and Co3) with electron configuration $t_{2g}^{6}e_{g}^{0}$, i.e., containing 4 empty 3d states. (**b**) Co${}^{2+}$ ion in the tetrahedral geometry (Co4) with electron configuration $e_{g}^{4}t_{2g}^{3}$, i.e., containing spin-up unpaired *d* electrons.

By performing orbital-projected density of states calculations, we are able to deduce the oxidation states of the Co atoms. In Figure 5, we present the projected DOS for the $3d_{xy}$, $3d_{zx}$, $3d_{zy}$, $3d_{z^{2}}$ and $3d_{x^{2}-y^{2}}$ bands. The DOS of Co atoms can be categorized into two groups, namely (i) the DOS of Co atoms in the octahedral sites, i.e., the trio of Co1, Co2 and Co3 and (ii) the DOS of Co4 located in the tetrahedral site. One can see that the orbital projected DOS for the two categories are different (Figure 3). Figure 5a–c, however, show that the XANES O pre-edge peak originates from contributions from $3d_{z^{2}}$ and $3d_{x^{2}-y^{2}}$ spin-up and spin-down orbital states of Co1, Co2 and Co3. This implies that the two orbitals $3d_{z^{2}}$ and $3d_{x^{2}-y^{2}}$ are empty, since they are predominant at the pre-edge peak. Thus, for Co1, Co2 and Co3, the plausible electron configuration for the *d*-shell is $3d^{6}$, thus suggesting that the three cobalt atoms are in the oxidation state +3. Furthermore, according to ligand field theory [30,31,32,33] for an octahedral coordination, the splitting of *d*-shells give rise to an electron configuration $t_{2g}^{6}e_{g}^{0}$ (see Figure 6a). This is because only the $e_{g}$ orbital participates in transitions occurring in the energy range containing the pre-edge peak (see Figure 5). Consequently, the Co${}^{3+}$ ions are not magnetic, since all *d* electrons are paired, as was previously also reported by Chen et al. [34], who also used the GGA + *U* approach. Similar conclusions were drawn in previous studies of electronic structure [29] and magnetization in Co${}_{3}$O${}_{4}$ [35].

In the case of cobalt atom Co4, the contributions to the pre-edge peak are due to spin-down $3d_{xy}$, $3d_{zx}$ and $3d_{zy}$ orbitals as shown in Figure 5d. Thus, the three orbitals $3d_{xy}$, $3d_{zx}$ and $3d_{zy}$ of spin-down are empty, since they contribute to the pre-edge peak. This result shows that the plausible electron configuration for the *d*-shell of cobalt Co4 is $3d^{7}$, which corresponds to the oxidation state +2. Furthermore, according to ligand field theory for a tetrahedral coordination, the cobalt atom Co4 has a high-spin nature, with the valence electron configuration $e_{g}^{4}t_{2g}^{3}$ as shown in Figure 6b. This is because only the $t_{2g}$ orbital of spin-down participates in the transition occurring resulting in the pre-edge peak (see Figure 5), as demonstrated experimentally by van Elp et al. [29]. Consequently, the Co${}^{2+}$ ions contain three unpaired *d* electrons, resulting in a magnetic moment of about 3.00 $\mu_{B}$ as a simple Hund’s rule for filling electronic shells in atoms would suggest. Indeed, the value of the Co${}^{2+}$ moment in Co${}_{3}$O${}_{4}$ has been reported as 3.02 $\mu_{B}$ in an earlier experimental work [35], and this magnetic moment is thought to be responsible for the magnetization in Co${}_{3}$O${}_{4}$ at low temperature, as reported by Chen et al. [34].

Finally, it is worthwhile to mention, albeit briefly, how our XANES data may find application in experimental catalysis studies involving the Co${}_{3}$O${}_{4}$ compound. The XANES spectra we have obtained corresponds to that of bulk and isolated Co${}_{3}$O${}_{4}$ system. However, when Co${}_{3}$O${}_{4}$ is exposed to reaction conditions during catalysis reactions, it is expected that changes will occur to the spectra shape and intensity. Thus, our calculated and experimental spectra provide the basis to follow the kinetic transformation of the Co${}_{3}$O${}_{4}$ under experimental reaction conditions while gaining insight into the structural and electronic changes happening to the constituent atoms making up the Co${}_{3}$O${}_{4}$ compound.

## 4. Conclusions

In conclusion, we have explored the electronic structures and deduced the magnetic properties of cobalt oxide Co${}_{3}$O${}_{4}$ by means of experimental and theoretical X-ray absorption near-edge structure (XANES) spectroscopy. We found good agreement between the calculated and the experimental spectra. The oxygen *K*-edge spectra show a strong pre-edge peak which can be ascribed to dipole transitions from O 1s to O 2p states which are hybridized with the unoccupied 3d states of cobalt atoms. Also, from the combined XANES and electronic structure analysis, we deduce the magnetic moments on the two types of Co atoms that are found in the Co${}_{3}$O${}_{4}$, i.e., Co${}^{3+}$ and Co${}^{2+}$. The former has zero magnetic moment while the latter has a spin moment of about 3.00 $\mu_{B}$. In fact, the magnetic structure of Co${}_{3}$O${}_{4}$ is due to antiferromagnetic ordering of the Co${}^{2+}$ spins, each of which is surrounded by four nearest neighbours with oppositely directed spins. Furthermore, we found that the contribution of Co${}^{2+}$ ions to the pre-edge peak is solely due to single spin-polarized $t_{2g}$ orbitals, while that of the Co${}^{3+}$ ions is due to spin-up and spin-down polarized $e_{g}$ orbitals. In summary, our work provides the background for further use of X-ray absorption spectroscopy to study nanostructured Co${}_{3}$O${}_{4}$ and its surfaces. Studies towards understanding the electronic processes underpinning adsorption and catalysis of reactive molecules on Co${}_{3}$O${}_{4}$ surfaces and the X-ray absorption analysis of active sites are in progress and the results will be presented in our future communication.

## Data Availability

Not applicable.
